# An investigation of left/right driving rules on deviations while walking

**DOI:** 10.1371/journal.pone.0186171

**Published:** 2017-10-11

**Authors:** Nicole A. Thomas, Owen Churches, Ian White, Christine Mohr, Yann Schrag, Sabrina Obucina, Michael E. R. Nicholls

**Affiliations:** 1 School of Psychology, Flinders University, Adelaide, Australia; 2 Institute of Psychology Universite de Lausanne, Lausanne, Switzerland; University of Toronto, CANADA

## Abstract

When traversing through an aperture, such as a doorway, people characteristically deviate towards the right. This rightward deviation can be explained by a rightward attentional bias which leads to rightward bisections in far space. It is also possible, however, that left or right driving practices affect the deviation. To explore this possibility, Australian (left-side drivers) and Swiss (right-side drivers) participants (n = 36 & 34) walked through the middle of an aperture. To control for the sway of the body, participants started with either their left or right foot. Sway had a significant effect on participants’ position in the doorway and the amount of sway was greater for Australians—perhaps due to national differences in gait. There was a significant rightward deviation for the Swiss, but not for the Australians. It is suggested that driving practices have a small additive effect on rightward attentional biases whereby the bias is increased for people who drive on the right and reduced in people who drive on the left.

## Introduction

Unilateral spatial neglect is a condition characterised by a reduced ability to attend to one side of space. In most cases, neglect occurs after damage to the parietal lobe of the right hemisphere, causing inattention to the contralesional hemispace [1]. As a consequence, when neglect patients perform tasks such as line bisection, they bisect the line to the right of its true centre, indicating a neglect of the left and over attendance to the right side of the line [2]. The failure of neglect patients to attend to the left side of stimuli extends to other clinical tasks including target cancellation [3, 4] and judgements of relative luminosity [5].

Neurologically healthy individuals also show subtle attentional biases. For stimuli located within reach (peripersonal space), a small, but consistent, leftward bias is observed which is termed pseudoneglect [6, 7]. When carrying out the line bisection tasks, healthy individuals transect the line slightly to the left of true centre. The leftward bisection bias is thought to be the result of a slight over-attendance to the left side of the line [2]. For stimuli located outside of reach (extrapersonal space), a rightward bias of attention has been observed [8, 9]. A dissociation between near and far space is supported by neurological research showing activation of the dorsal (intraparietal sulcus) and ventral (medial temporal cortex) streams for the bisection of lines placed in near and far space, respectively [10].

Pseudoneglect can affect everyday interactions within the environment. For example, Turnbull and McGeorge [11] found that participants tended to recall more collisions on their right-hand side. Nicholls, Loftus, Mayer, and Mattingley [12] followed up with a study examining the occurrence of leftward and rightward collisions under laboratory conditions. Participants were asked to walk through a narrow doorway and the number of bumps to each side of the doorway was recorded. More rightward collisions were observed, and this bias was exaggerated when the left hand (therefore right hemisphere) was active and diminished when the right hand (therefore left hemisphere) was active. Nicholls et al. [2007] therefore replicated the rightward bias in collisions reported by Turnbull and McGeorge and demonstrated that the effect was driven by asymmetries in hemispheric activation. Asymmetries in navigation also occur for non-ambulatory tasks, such as wheelchair navigation [13].

To explain the rightward navigation asymmetry, Nicholls et al [13] and Robertson, Forte, and Nicholls [14] developed a model of attentional asymmetry based on line bisection. Because the doorway or aperture is located outside of reach, Nicholls reasoned that participants would estimate the centre of the door slightly to the right of true centre (see [8]). Given that Berti et al. [15] demonstrated that participants mentally ‘mark’ the centre of a target and then head towards that point in a ballistic fashion, a rightward bias in bisection would be expected to cause participants to pass to the right of true centre in a doorway. In support of this proposition, Robertson et al. [14] found that participants looked to the right of centre from the beginning to the end of trials during a remote wheelchair navigation task. Furthermore, Nicholls, Jones and Robertson [16] found that rightward bisection biases for a line placed in far space were related to the subsequent position of a remote-controlled miniature car as it passed through a doorway.

While the results discussed above are consistent with an attentional account of navigational biases, alternative explanations have also been proposed. For example, national differences related to the side of the road on which people drive could affect navigation asymmetries. There is anecdotal evidence that driving habits affect the side on which pedestrians pass one another (see: https://en.wikipedia.org/wiki/Pedestrian_etiquette). It is therefore plausible that driving habits could cause, or at least moderate, navigation asymmetries.

Some insight into the effect of driving practices on navigation asymmetries can be gained by comparing experiments that have used left- and right-side drivers. In relation to left-side drivers, rightward biases have been reported by Nicholls and colleagues for ambulatory [12, 17], wheelchair [13, 14] and miniature car [16] tasks in Australian drivers. In Japan, where drivers also drive on the left, weak evidence of a rightward bias was observed for an ambulatory task (Expt. 1)–but this was affected by the foot used to start walking (elaborated further below, [18])

For right-side drivers, a rightward deviation has been reported by Jang et al. [19] for Koreans when controlling a car in a simulator. A rightward bias has also been reported for a virtual route-following task for a group of North Americans. This bias, however, was limited to the upper hemispace—and reversed to a leftward bias when in the lower hemispace [20]. More leftward collisions have been reported by Hatin et al., [21] for an ambulatory task in a North American population. Finally, Cinelli, Patla and Allard [22] required participants to pass through a doorway, which opened and closed at a number of different frequencies. Cinelli et al. [22] found that participants looked towards the left more often and suggested that their North American population were accustomed to passing cars and people to their left and therefore pay more attention to this side.

As can be seen, between-experiment data related to driving direction are not clear. It is difficult to make meaningful comparisons, however, given the wide range of methodologies that have been used. To address this issue, the current study tested left- and right-side drivers within the one experiment using the same methodology. Left- and right-sided drivers were sampled from Australia and Switzerland, respectively. A number of hypotheses can be proposed. It is possible that driving on the left causes rightward deviations—perhaps to avoid the curb. In which case, a rightward deviation should be observed for Australians—but not the Swiss, Alternatively, and perhaps more plausibly, driving habits cause a bias to the ipsilateral side. In which case, the rightward deviation may be strongest for right-side drivers (Swiss) and reduced in left-side drivers (Australians).

Another alternative explanation for navigation asymmetries is the foot used to start walking. While starting-foot cannot explain the asymmetries observed for navigating a wheelchair [13, 14] or a miniature car [16], it could play a role in ambulatory studies. Fujikake et al. [18] proposed a motor asymmetry model that incorporates the sway of the upper-body mass as people walk. Specifically, when stepping with the left, an individual’s centre of mass shifts over the left foot, then as the next step is taken, it shifts above the right foot—creating a swaying motion of the upper body. Fujikake et al. argued that the predominance of rightward collisions for ambulatory tasks in previous research were a function of the lead foot used to step through the doorway. The experiments by Nicholls et al. [12, 17] used a set starting distance from the doorway and did not control for starting-foot. Therefore, since most right-handed individuals prefer to step off with their right foot [23], the distance at which participants started from the doorway may have resulted in a systematic asymmetry in body sway as the participant walked through the door.

Given the potential importance of body sway on ambulatory navigation asymmetries, the current study sought to control for these factors. Unlike Fujikake et al. [18], we did not adjust the starting position to match the length of the participants’ strides. Instead, the Australian and Swiss participants were asked to start trials with either their left or right foot at fixed starting positions. Although this technique does not allow us to know exactly which foot was leading as the participant entered the doorway, it does systematically change the foot that would have lead into the doorway. Therefore, although the direction of the effect cannot be predicted, if leading foot is important to asymmetries in ambulatory navigation, there should be an effect of starting-foot. The distance between the starting position and the doorway was also manipulated between 1.8 and 2.1m. The difference between the starting position was designed to be roughly half of one stride. If leading foot is important, as suggested by Fujikake et al., starting distance should affect the position of participants as they pass through the doorway.

## Method

### Participants

Students from Flinders University in Australia (f = 24, m = 12, mean age = 22.2 years, SD = 3.22 years) and Universite de Lausanne in Switzerland (f = 33, m = 1, mean age = 21.5 years, SD = 3.77 years), participated in the experiment for either course credit or payment. The minimum driving age in Switzerland and South Australia is 18 and 16 years, respectively. Although we did not ask participants if they drove, all participants were old enough to drive and would have been familiar with the side of the road on which they drove. All participants achieved a positive score (M = 9.22, SD = 1.73) on The Flinders Handedness Survey (FLANDERS; [24]), confirming the absence of any left-handed individuals. Left-handers were excluded from the study because they are known to have a weaker right hemisphere specialisation for spatial tasks, such as line bisection [25]–and may therefore weaken asymmetries in navigation. Participants were asked to report if they had normal movement in relation to walking and whether they had normal or corrected-to-normal visual acuity. No participant reported any motor, neurological or visual issue that could affect their ambulatory ability. Although subclinical vestibular impairment could impact body sway, this function was not assessed in the current study as it is unlikely to affect many individuals in a healthy undergraduate population. This research complied with the American Psychological Association Code of Ethics and was approved by the Institutional Review Boards of Flinders University and the University of Lucerne. Informed consent was obtained from each participant.

### Apparatus

**Fig 1:**
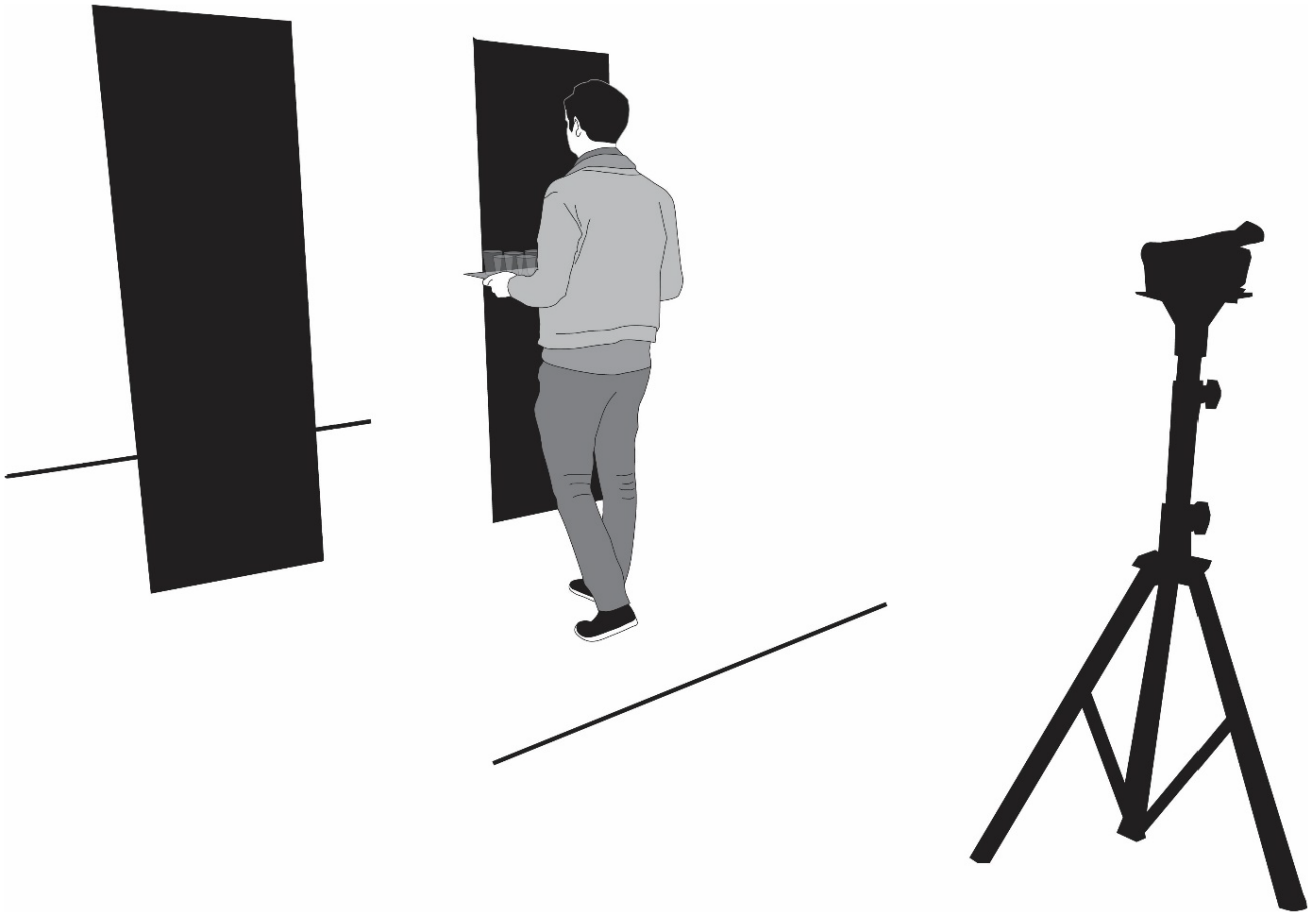
Illustration of the experimental set-up sowing a participant walking towards the aperture carrying a tray of drinks.

A doorway was created using two frames (2160mm high by 800mm wide). To increase the salience of the aperture, black cloth was used to cover the frames giving the appearance of a doorway in a solid wall (see Fig 1). An infrared sensor was attached to the inside of the doorway frame creating a beam across the aperture. The sensor was connected to a red LED mounted on the top right corner of the doorway frame (out of the participant’s view). A digital HD video camera (model: Sony HDR-PJ430VE) was set up on a tripod behind the participant directly in line with the centre of the doorway at a distance of 3.1m and height of 1.2m to record their position in the doorway as the LED flashed. To engage the arms in a symmetrical activity and to obscure the main aim of the experiment, a balancing task was used in which participants carried six plastic cups filled with water to 20mm from the brim placed evenly on a rectangular tray with both hands. Previous research by Nicholls and colleagues [12] has also used a concurrent task and it is likely that the extra cognitive load and distraction may play a role in the development of attentional asymmetries [26]

### Procedure

The experiment was conducted in a room cleared of any asymmetrical distractions. The position of the doorway, camera tripod and two start/finish points were measured and marked on the floor. The camera position was calibrated prior to testing each time to check that its midpoint was in line with the centre of the doorway to ensure accurate measurement of the dependent variable. The width of the doorway was set at 800mm so that participants were able to comfortably fit through without any compensation to normal walking posture (e.g. turning of shoulders).

Participants were asked to pick up the tray of drinks using both hands, tuck their elbows at their sides and hold it along the midpoint of their body. They were asked to refrain from spilling any of the water to increase the salience of this part of the task. Participants were positioned facing the doorway with their feet together up against the starting-line. They were then asked to start walking with either the left or right foot (depending on condition) and continue at a normal pace through the doorway to the stopping line as indicated by the experimenter. Because most people have a right foot preference for stepping-up and walking-off [23, 27], starting-foot was controlled within participants to avoid an asymmetry based on idiosyncratic foot preferences. As participants passed through the doorway they broke the infrared beam causing the LED to flash—thus providing a uniform and accurate measure of when they entered the aperture.

On reaching the finish line, participants were asked to return back through doorway and repeat the process from the same starting point using the same starting foot until notified by the experimenter. High definition video footage was shot continuously for each participant until they had completed all trials. Each participant completed four conditions: 1.8m start left foot, 1.8m start right foot, 2.1m start left foot, and 2.1m start right foot. The stopping point for each condition was the distance beyond the doorway identical to the starting position, e.g. for the 2.1m starting distance participants were asked to stop at a point 2.1m past the aperture. Each participant completed 4 blocks of 10 trials with order balanced between participants with distance always together.

The point at which participants passed through the aperture was determined by reviewing the recorded footage using VLC media player 2.1.3. and pausing when the LED flashed indicating the subject had broken the infrared beam. A snapshot of the paused footage was taken for each trial and the dependent variable was ascertained by measuring the distance from midpoint of the neck along the coronal plane to the inside of the left-hand doorpost. Measurements were made in pixels using GIMP 2.8.10 GNU Image Manipulation Program and then converted to millimetres using the known physical distance between the door-posts. These raw measurements were transformed by subtracting half of the doorway width (400mm) thus giving negative and positive values in mm reflecting deviations to the left and right respectively from the centre of the doorway.

To ensure the reliability of our scoring method, an independent scorer blind to the aims of the study scored the data from two randomly selected participants. Comparison between the experimenter’s and independent scorer’s measurements of the dependent variable using Cronbach’s alpha revealed a high level of inter-rater reliability (α = 0.99). Importantly, methods such as this have shown strong test-retest reliability [28].

### Statistical analysis

Shapiro-Wilk tests revealed that the data were normally distributed and parametric tests were therefore used. The data were initially tested using a series of one-sample t-tests to determine which conditions produced a statistically significant deviation away from zero (the middle). The deviation data were then analysed with a mixed model ANOVA with distance (1.8m, 2.1m) and starting-foot (left, right) as within participant factors and Country (Australian, Swiss) as a between participant factor. Effect size is represented by the Eta square value. Mauchly’s test of sphericity revealed that the assumptions of the ANOVA were not violated. Although no predictions were made in relation to the effect of sex, an analysis was carried out using the same ANOVA model, but with sex (m,f) as an additional factor. The results of this analysis were essentially the same as the simpler analysis reported here. There was no main effect of sex and sex did not interact with any factor. In addition, the status of all significant and non-significant effects remained the same irrespective of whether sex was included in the model. We therefore report the results of the simpler ANOVA model.

## Results

### One sample t-tests

**Fig 2:**
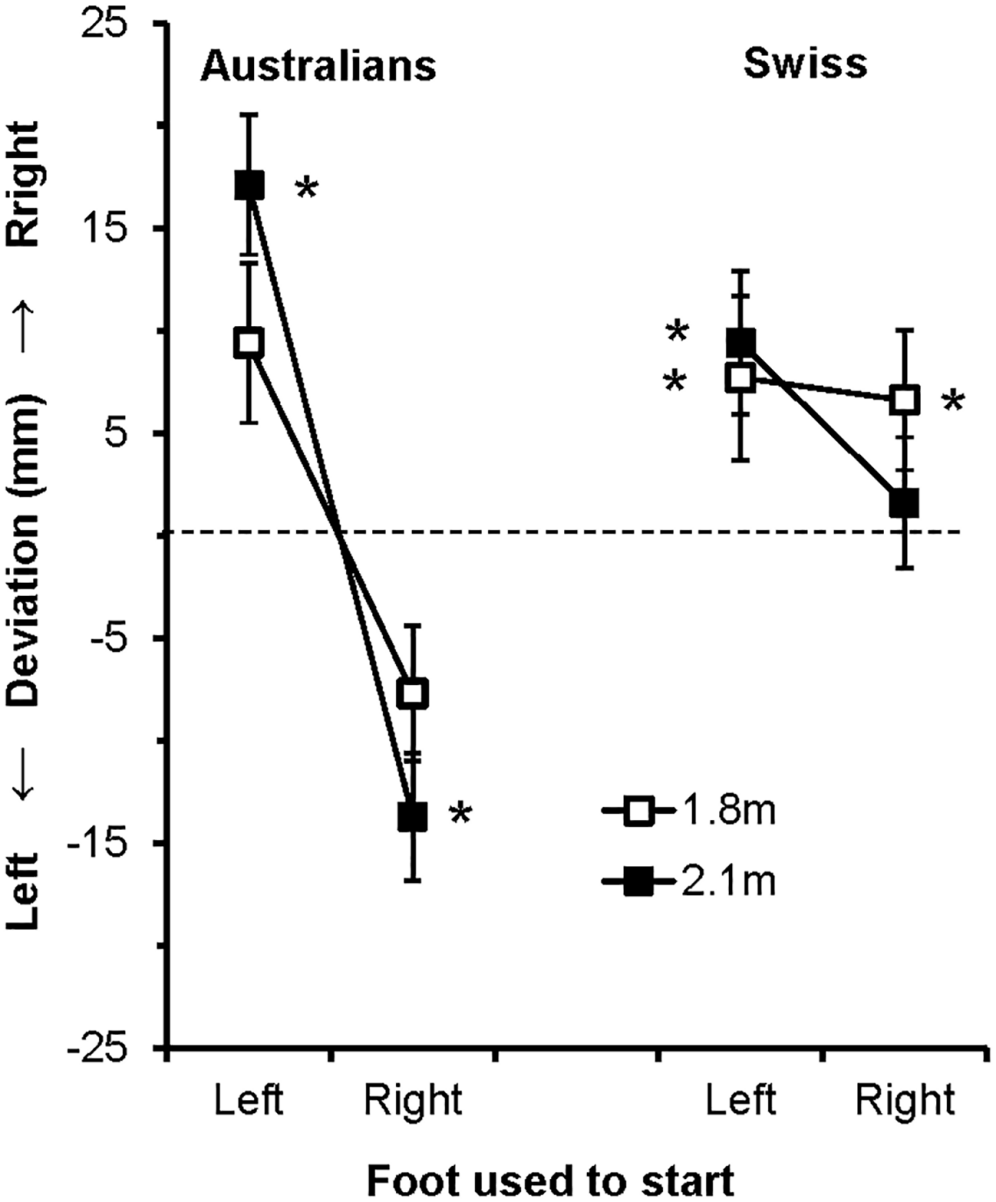
Graph showing mean deviations for the Australian and Swiss populations for the left- and right-start conditions at starting distances of 1.8 and 2.1m. Negative and positive scores indicate that participants passed to the left and right of centre, respectively. The error bars show the standard error.

When the data were collapsed across all conditions and groups, there was a significant bias of 3.7mm to the right of true centre [t(69) = 2.255, p = .027]. When the data were broken down by country, the rightward deviation failed to reach significance for the Australians [$\bar{x}=1.277\text{mm}$, t(35) = .422, ns], but was significant for the Swiss [$\bar{x}=6.374\text{mm}$, t(33) = 5.716, p < .001]. Finally, each of the four conditions within the Australian and Swiss populations was examined. The critical p value was adjusted for multiple comparisons to p < .006. The conditions that were significantly different from zero are marked with an asterisk in Fig 2. As can be seen from the figure, there were significant rightward and leftward biases in the 2.1m condition for the Australians. The Swiss data showed significant rightward biases for all conditions except the 2.1m right-start condition.

### ANOVA

While there was no statistically significant effect of distance [F(1,68)<1], there was significant effect of starting-foot [F(1,68) = 44.110, p < .001, *η*_*p*_^*2*^ = .393] reflecting a stronger rightward deviation in the left-start condition compared to the right-start condition. There was also a significant interaction between distance and starting-foot [F(1,68) = 7.115, p = .010, *η*_*p*_^*2*^ = .095]. Fig 2 shows that the effect of starting-foot was stronger for the 2.1m trials compared to the 1.8m condition. Post-hoc tests revealed that, while the effect of starting-foot was significant for both distances, the effect was markedly weaker in the 1.8m condition [t(70) = 2.990, p = .004] compared to the 2.1m condition [t(70) = 6.465, p < .001].

In relation to the between participant factor, there was no main effect of Country [F(1,68) = 2.839, p = .127, *η*_*p*_^*2*^ = .034]. There was, however, a significant interaction between Country and starting-foot [F(1,68) = 20.631, p,.001, *η*_*p*_^*2*^ = .233]. As can be seen from Fig 2, the effect of starting-foot was much stronger for the Australians compared to the Swiss. Post-hoc tests supported this inference by showing a strong effect of starting-foot for the Australians [F(1,35) = 43.310, p < .001, *η*_*p*_^*2*^ = .553] and a marginally significant effect for the Swiss [F(1,33) = 4.426, p = .042, *η*_*p*_^*2*^ = .118]. No other interactions approached statistical significance.

## Discussion

The current study examined the effect of national driving practices on ambulatory asymmetries when passing through a doorway. Overall, there was statistically significant bias towards passing through the doorway to the right of true centre. This bias is consistent with the model proposed by Nicholls et al [13] and Robertson, Forte, and Nicholls [14], which proposes a bisection asymmetry caused by right hemisphere specialisation for spatial attention. When the data were broken down by country, however, one sample t-tests revealed a significant rightward deviation for the Swiss, but not for the Australians. Given that a rightward bias has reliably been observed by Nicholls et al. [12, 17] for Australians during an ambulatory task, it is not clear why a rightward bias did not emerge in the current study. It is well known that interindividual differences exist in the direction and degree of pseudoneglect [29, 30]. It is therefore possible that, by chance, the current study included a relatively higher proportion of Australians with reversed attentional asymmetries. The current study also used a slightly different methodology to that used by Nicholls et al. [12, 17]. In the original studies, the aperture was set so that it was the same width as the participant—and the number of hits to the left and right were compared. In the current study, the aperture was set much wider and the participant’s position with respect to the middle was measured. Although this latter technique has proved successful in tasks requiring wheelchair [13, 14] and miniature car [16] navigation, it is possible that the technique is less sensitive for ambulatory studies where participants are not concerned with colliding with the side of the aperture.

The effect of leading foot was controlled by varying starting foot and distance. In relation to starting foot, the rightward bias was strongest when the participant started with their left foot and weakest, or reversed when participants started with their right foot. The effect of starting foot is consistent with the results reported by Fujikake et al. [18]. Because we did not measure interindividual differences in stride length and match the starting position to suit this, we do not know exactly which foot was used as participants entered the aperture. However, Fujikake et al. [18] reported that their average starting distance was 2.0m, which allowed four steps to be taken. In addition, research by Das, Dhume and Iyer [31] shows that the average stride length when carrying a load is approximately 600mm. Given that the foot was behind the starting line (and the foot is approximately 300mm long), this means that roughly four full strides would be taken as the participant entered the doorway. The left-start condition would have therefore placed the right foot in the doorway and vice versa for the right-start condition. This foot placement would have resulted in a shift of the upper torso ipsilateral to the foot that was being placed down. The results are therefore consistent with the idea that body sway affects the position of the upper torso as participants carry out an ambulatory aperture navigation task [18].

There was also a significant interaction between starting-foot and distance. For both countries, the effect of starting-foot was stronger for the 2.1m condition compared to the 1.8m condition. As outlined above, although we don’t know the exact position of the foot as it entered the doorway, we can estimate that four full strides were made on average between the 2.1 starting position and the doorway. Because the left or right foot was being placed down as the participant entered the doorway, one would predict the maximum sway at this point—producing the largest effect of starting foot. In contrast, the 1.8m starting position would have placed participants, on average, somewhere mid-stride as they passed through the doorway, which reduced the effect of body sway at that point. The effect of starting-foot is therefore consistent with the sway theory proposed by Fujikake et al. [18].

The effect of starting-foot was much stronger for the Australians compared to the Swiss. Indeed, for the Australians, the reversal of the deviation as a function of foot is quite similar to that observed by Fujikake et al. [18] in experiments 2 and 3. The Swiss show a more subtle effect of starting-foot with no sign of a reversal in deviation between the left- and right-start conditions. While a difference in nationality in the effect of starting-foot was unexpected, it may make sense. A number of studies have investigated national differences in gait. For example, Ryu, Choi, Choi and Chung [32] compared gait characteristics between Koreans and Western people. They found that Koreans took shorter strides and had smaller hip movements in the sagittal and frontal plane than their Western counterparts. Al-Obaidi, Wall, Al-Yaqoub and Al-Ghanim [33] tested basic gait parameters between Kuwaitis and Scandinavians and found differences in speed and step-length that interacted with sex. Even within Europe, differences in gait velocity have been observed between rural residents in Austria and urban residents of Germany [34]. It is therefore clear the national differences in gait need to be taken into account in any cross-national study investigating of gait. While there are no data relating to differences in gait between Australians and Swiss, it is possible that Australian walk with grater lateral hip movement or swagger. National differences in movements such as this would produce greater sway in the Australian population compared to the Swiss and therefore produce the larger effect of starting-foot observed.

A reviewer raised the important possibility that the difference between Australians and Swiss in the effect of starting-foot is related to differences in sex ratios between the samples. For Australians, 66% of the sample was female and this rose to 97% for the Swiss sample. Because, on average, women are shorter than men, it seems reasonable that women will also take shorter strides [35]. It is therefore possible that the difference in the effect of starting-foot between the counties is caused by the higher proportion of women in the Swiss sample, who take shorter strides. This proposition was tested by including sex as an additional between-participants factor in the ANOVA model. Because the analysis showed that sex had no main effect and did not interact with any other factor, the potential role of sex in the current study can be dismissed.

Finally, the analyses revealed no statistically significant overall difference between the Australians and Swiss in the magnitude of the rightward deviation. That said, Fig 2 appears to show that the rightward deviation was stronger in the Swiss compared to the Australians. In addition, as noted in the beginning of this discussion, the rightward deviation was significant for the Swiss, but not for the Australians. The results are therefore partially consistent with the proposal that driving practices cause a navigation asymmetry ipsilateral to the side on which they normally drive. Thus, there may be a resting attentional bias driven by functional specialisation of the cerebral hemispheres, which causes individuals to deviate to the right when bisecting an aperture. Driving habits have an additive effect on this baseline whereby the rightward bias is increased by right-side drivers (Swiss) and reduced in by left-side drivers (Australians). While the effect of driving habits failed to reach statistical significance in the present study, it would be interesting to follow up this ambulatory study with motorised vehicles where the effect of driving practices may be stronger.

A number of limitations in the present study could be addressed in future research. by providing a more detailed analysis of participant’s gait. To exclude the possibility that participants’ height, weight and body mass affects gait parameters, these variables should be measured and controlled. In addition, the study could benefit be identifying participants’ individual stride length and then using this to adjust the starting point for each participant. Finally, a movement analysis technique, which accurately records the position of various body parts as participants walk towards and through the door would be the ideal method of testing asymmetries in navigation.

## Supporting information

S1 File Full data file of all participants. (SAV)
